# The Mycetoma Knowledge Gap: Identification of Research Priorities

**DOI:** 10.1371/journal.pntd.0002667

**Published:** 2014-03-27

**Authors:** Wendy W. J. van de Sande, El Sheikh Maghoub, Ahmed H. Fahal, Michael Goodfellow, Oliverio Welsh, Ed Zijlstra

**Affiliations:** 1 ErasmusMC, Department of Medical Microbiology and Infectious Diseases, Rotterdam, The Netherlands; 2 Mycetoma Research Centre, University of Khartoum, Khartoum, Sudan; 3 School of Biology, University of Newcastle, Newcastle upon Tyne, United Kingdom; 4 Dr. Jose E. Gonzalez University Hospital, Universidad Autónoma de Nuevo León, Department of Dermatology, Ave Madero y Ave Gonzalitos, Colonia Mitras Centro, Monterrey, Nuevo Leon, Mexico; 5 Rotterdam Centre for Tropical Medicine, Rotterdam, The Netherlands; Fundação Oswaldo Cruz, Brazil, United States of America

## Abstract

Mycetoma is a tropical disease which is caused by a taxonomically diverse range of actinomycetes (actinomycetoma) and fungi (eumycetoma). The disease was only recently listed by the World Health Organization (WHO) as a neglected tropical disease (NTD). This recognition is the direct result of a meeting held in Geneva on February 1, 2013, in which experts on the disease from around the world met to identify the key research priorities needed to combat mycetoma. The areas that need to be addressed are highlighted here. The initial priority is to establish the incidence and prevalence of the disease in regions where mycetoma is endemic, prior to determining the primary reservoirs of the predominant causal agents and their mode of transmission to susceptible individuals in order to establish novel interventions that will reduce the impact of the disease on individuals, families, and communities. Critically, economical, reliable, and effective methods are required to achieve early diagnosis of infections and consequential improved therapeutic outcomes. Molecular techniques and serological assays were considered the most promising in the development of novel diagnostic tools to be used in endemic settings. Improved strategies for treating eumycetoma and actinomycetoma are also considered.

## Introduction

Currently there are 17 infections listed by the World Health Organization as neglected tropical diseases and three others are cited as neglected conditions [1]. Until July 2013, mycetoma was absent from the WHO list, even though its estimated prevalence of two per 100,000 inhabitants was comparable to that of other recognized neglected diseases, such as Buruli ulcer and Human African trypanosomiasis [2], [3].

Mycetoma is an implantation mycosis [4], characterized by large tumor-like swellings and located mainly in the extremities. It can be caused by taxonomically diverse microorganisms, both of bacterial (actinomycetoma) and fungal origin (eumycetoma) [5]. The most common causative agents include the fungus *Madurella mycetomatis* and the actinomycetes *Nocardia brasiliensis, Actinomadura madurae, Streptomyces somaliensis*, and *Actinomadura pelletieri* [3]. The disease is mainly found in tropical and subtropical regions of the world, and the majority of patients are reported from Mexico, Senegal, Sudan, and India, but its true prevalence and incidence are not well defined [3]. Furthermore, there are no rapid diagnostic tools, while treatment, especially for eumycetoma, is unsatisfactory, resulting in high morbidity, including amputation of limbs (submitted; see Acknowledgments).

On February 1, 2013, a landmark meeting was held in Geneva, Switzerland that was attended by experts on mycetoma from around the world. Its aim was to review all currently available information and to identify knowledge gaps and research priorities. It was concluded that basic epidemiological information is lacking: it is not known how many people are suffering from mycetoma or where the disease is most prevalent. Furthermore, early detection of mycetoma is difficult, while treatment is far from satisfactory for eumycetoma patients.

In this paper, we consider the knowledge gaps and research priorities identified at the Geneva meeting.

## Epidemiology

### The incidence, prevalence, and mapping of mycetoma

Although mycetoma was first introduced to modern science in 1694 by Kaempfer in his dissertation [6], it is still not known how many people are affected by this disease. In order to get a rough estimation on the global burden of mycetoma, van de Sande performed a meta-analysis in which 8,763 cases were reviewed [3]. These cases were from various countries, including India, Mexico, Niger, and Sudan [7]–[16]. By dividing the number of reported cases by the country population in each year, an estimate of the prevalence per country was calculated. The estimated prevalence ranged from 3.49 cases per 100,000 inhabitants in Mauritania to <0.01 cases per 100,000 inhabitants in many other countries [3]. The estimated prevalence for the endemic areas of mycetoma, Mexico and Sudan, were 0.15 and 1.81 cases per 100,000 inhabitants, respectively. Although this study gave insight into the prevalence of mycetoma, the estimates do not reflect the magnitude of the problem. The total number of patients is probably much higher, since the prevalence was mainly derived from single-center studies, and large epidemiological studies from a number of countries in which mycetoma is known to exist, such as South Africa, are lacking [3], [17]–[19]. Furthermore, at the Mycetoma Research Centre in Khartoum, more than 6,400 patients have been seen and treated, but this has not been reported in the literature (Fahal, in preparation). Surveys performed in mycetoma-endemic villages in Sudan suggest a prevalence ranging from 0–8.5 per 1,000 inhabitants (Table 1) [20], [21].

**Table 1:** Prevalence of mycetoma as reported in Sudanese villages.*

Village	Year	Population	Number of cases	Prevalence per 1,000	Reference
Abu Gumri	1960	1,300	8	6.2	[20]
El Andalous	2011	2,835	24	8.5	[21]
Arkawit	1960	56	0	0	[20]
Beriab	1960	1,350	7	5.2	[20]
Denegila	1960	1,025	1	1.0	[20]
Rabua	1960	500	1	2.0	[20]
Seraam	1960	500	0	0	[20]

*Data obtained mainly from [20].

It can be concluded from the current situation that (inter)national surveillance programs are needed to get a reliable indication of the prevalence of mycetoma. In the first instance, this could be achieved by using a surveillance form similar to the BU02 (http://www.who.int/buruli/control/ENG_BU_02.pdf), which is used for Buruli ulcer [3]. The BU02 form is used to establish the prevalence of Buruli ulcer in different districts in Cameroon and the Democratic Republic of Congo (DRC). The Akonolinga district in Cameroon and the Songololo territory in DRC were found to have a high prevalence [22], [23]. Subsequently, Buruli ulcer control projects launched in these two locations resulted in an increase in the number of Buruli cases detected [22],[23]. As a result, there was a significant increase in the proportion of early lesions and simple ulcerative forms and a decrease in the proportion of relapse cases [23]. These results indicate that defining areas with a high prevalence and establishing informed local health institutes reduces the burden of disease. The application of a similar approach for mycetoma, including the use of standardized forms and the establishment of national reference centers, can be expected to generate more reliable data on the incidence and prevalence of mycetoma. In Sudan, part of the infrastructure and data needed are already available. A national Mycetoma Research Centre is situated in the capital, Khartoum. Furthermore, in 1956, Abbott already demonstrated that in Atbara, Ed Dueim, and Wad Medani the prevalence among hospital admissions was higher than in the capital (prevalence 9.2, 9.3, and 11.8 per 1,000 hospital admissions, respectively, versus 5.1 in Khartoum) [8]. The distance from these cities to Khartoum ranges from 190 km to 328 km, making the trip to the Mycetoma Research Centre difficult and expensive. The late presentation to the clinic is attributed to the nature of mycetoma, which is usually painless and slowly progressive, and the lack of health education. Furthermore, since most of the patients are of low socioeconomic status, financial constraints also play an important role. Therefore, more patients will be reached when regional health centers with healthcare personnel trained in the disease are established in highly endemic regions. The need was partially realized in October 2012 when a new Mycetoma center was opened in Wad Medani [24]. However, more satellite centers are needed in a country as large as Sudan. Better incidence and prevalence data are necessary to determine appropriate sites for new mycetoma institutes in the Sudan and other mycetoma regions, including India, Mexico, and Senegal.

## The Mode of Transmission

**Figure 1:**
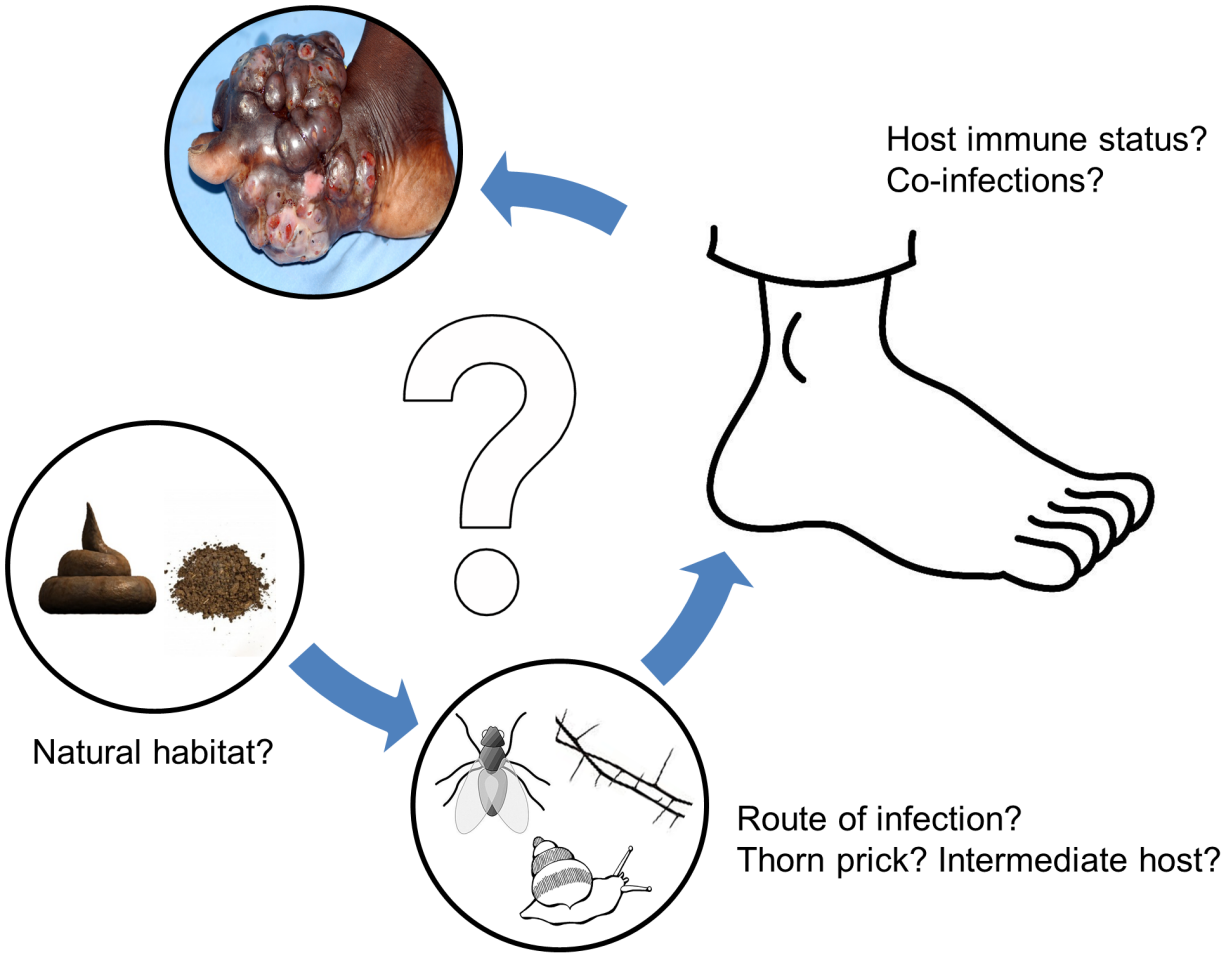
Possible hypothesis for mycetoma infection route. At the moment, many questions still remain about the various steps in the development of mycetoma. First, it is not known where the causative agents reside. What is the natural habitat of these agents? Both soil and dung have been implicated, but other niches could also be involved. Second, how is the causative agent introduced into the subcutaneous tissue? Is it via a thorn prick as implicated for years, or are vectors, such as insects, involved? When the causative agent is introduced in the subcutaneous tissue, will it always cause mycetoma, or does the host play a role as well? Do immune status, genetic background, or co-infections play a role in the development of mycetoma or even the extent of the infection?

Other information which is lacking is how people become infected with the causative agents of mycetoma. The primary reservoir of the causal agents is believed to be soil. Among the actinomycetes, *A. madurae* [25], *A. pelletieri* [25], *N. asteroides* [25]–[27], *N. brasiliensis* [25], [28], *S. somalienis* [25], [26], and among the fungi, *Falciformispora senegalensis* (previously known as *Leptosphaeria senegalensis*) [25], *M. mycetomatis* [29], *Neotestudina rosatii* [25] and *Scedosporium boydii* [30]–[32] have been cultured from soil, though in many cases it is difficult to be certain that strains have been correctly identified, especially since they tend not to have been properly preserved. Several recent attempts to culture the fungus *M. mycetomatis* from soil have failed, although DNA of the organism was detected in 17 out of 74 soil samples and in one out of 22 thorn samples [33]. There is now evidence based on phylogenetic analyses that *M. mycetomatis* may be closely related to dung-inhabiting fungi, suggesting that its primary reservoir could overlap with the natural niche of these fungi [34]. Another mycetoma-causative agent, *N. asteroides*, has been isolated from cow dung in India [26]. More studies are needed to establish the environmental niches of the mycetoma causative agents and their mode of transmission to patients (Figure 1).

Irrespective of the natural niche of the causative agent, it has to reach the subcutaneous tissue in order to cause mycetoma. Historically, this disease has been associated with minor trauma caused by thorn pricks, stones, or snake and insect bites [35]. Also, how the grain itself forms has not been studied. Some studies have focused on the components of the grain but the formation itself is not understood [36]–[39]. The detection of remnants of thorns inside lesions of mycetoma patients has given credence to the importance of thorns [35], especially since thorns are abundant in mycetoma-endemic regions. *F. senegalensis* and *Medicopsis romeroi* (previously known as *Pyrenochaeta romeroi*) have been isolated from thorns [35]. However, thorn pricks are also common in people who do not develop mycetoma, a result which suggests that other factors may be important and/or that the immune system has a role in the disease process.

The role of the immune system in mycetoma patients has been studied to some extent [40]–[43]. It would seem that there are no obvious defects in the immune system, but some single nucleotide polymorphisms have been linked with mycetoma development [41], [42]. Furthermore, from animal experiments it appears that a Th2-response might be necessary to induce mycetoma [44]–[46]. Recently it was demonstrated that a co-infection with schistosomiasis, a strong Th2-inducer, was linked to mycetoma susceptibility [47]. The role of animals should be investigated in endemic areas where people live in close contact with animals (cattle, donkeys, dogs, sheep, chicken, etc.) that are kept in compounds and where the ground is covered with animal dung. The role of animal reservoirs has been shown to be important in the development of many other (subcutaneous) infections, as exemplified by plague [48], schistosomiasis [49], Lyme disease [50], and leishmaniaisis [51]. For mycetoma, intermediate hosts have not been detected, though, interestingly, *N. asteroides, N. otidiscaviarum*, and *S. somaliensis* have been isolated from the gut and casts of four different earthworm species [26].

Identifying the primary reservoir and the route of infection could help in developing control strategies to prevent transmission of the causative agent to the subcutaneous tissue (Figure 1). Examples of such control measurements for other infectious diseases are numerous, ranging from mass drug administration to combat schistosomiasis [52] to distributing bed nets for the control of malaria [53]. The identification of risk factors associated with the development of mycetoma could provide clues to the natural route of infection. Epidemiological screening and culturing of causative agents from habitats in endemic regions is needed to answer these questions and plan effective controls. Easy interventions such as the distribution of closed shoes, removal of cattle and other animals from the compound, or clearing thorny bushes from the compound's immediate surroundings could possibly prevent mycetoma.

## Development of Methods for Early Diagnosis

In order to be able to determine the true burden of mycetoma and to identify the primary reservoir(s) and potential intermediate host(s) of its causative agents, good, reliable, fast, and cheap diagnostic tools are needed. At present, it is very difficult to reliably discriminate between infected and noninfected individuals in the field (submitted; see Acknowledgments); early cases without lesions are almost impossible to identify since they don't show classical signs of mycetoma and grains are still absent. The available diagnostic tools include imaging to determine the extent of lesions along different tissue planes, and cytological, histopathological examinations and culturing of grains to identify the causative agent (submitted; see Acknowledgments). Deep surgical biopsy material is needed for the latter two procedures. Identification of causal agents to the species level with histology is next to impossible, while culturing them is difficult and time-consuming, especially for nonsporulating fungi (submitted; see Acknowledgments). Misidentification of causal agents in the past has caused poor therapeutic outcomes [54]. More reliable diagnostic techniques depending on DNA analysis have been developed for the detection of actinobacterial and fungal causal agents, but such approaches are too expensive for use in endemic areas at the present time [55], [56]. Therefore, one of the first priorities is to develop fast and reliable diagnostic tools which can be used as point-of-care tests in regions where mycetoma is endemic. Since it has been demonstrated that 65% of all mycetoma cases can be attributed to *M. mycetomatis, N. brasiliensis, A. madurae, A. pelletieri, N. asteroides*, and *S. somaliensis*, the initial focus should be on the identification of these causative agents. Diagnostic methods which are most applicable are DNA-based identification tools such as loop-mediated isothermal amplification (LAMP), and serological assays.

LAMP has been successfully used in Africa and India [57]–[59] to diagnose Human African trypanosomiasis (HAT), malaria and visceral leishmaniasis. Sufficient DNA can be isolated from a dried blood spot on Whatman filter paper using simple extraction methods [58]–[60]. For the detection of Buruli ulcer, swabs or fine needle aspirates are used [61]. Isolated DNA can be amplified by using a heating bath and the resulting amplicons are usually visualized by adding (fluorescent) dyes [57]–[61]. This technique might be adapted for mycetoma, and various patient samples, including blood, serum, or fine needle aspirates, can be tested to determine the specificity and sensitivity of this assay in mycetoma patients.

Other diagnostic tools applicable in endemic settings are serological-based procedures, such as latex bead agglutination assays and dipsticks. By binding either an antigen [62], [63] or a specific antibody [64], [65] of the causative agent to a latex bead, a cheap and fast screening tool for serum can be developed that minimally requires the use of any equipment. Latex bead assays have been developed for other tropical infections, including visceral leismaniasis [62], [64] and paracoccidioidomycosis [63]. In order to develop a specific agglutination assay, a discriminatory antigen needs to be selected. Whole genomes are available for *N. brasiliensis* [48] and *S. somaliensis* [47], while those of other causal agents such as *M. mycetomatis* and *S. sudanensis* are being generated. The genomes of these species will be ideal resources to select specific proteins for rapid serodiagnosis.

## Treatment

Accurate diagnosis of the causal agents of mycetoma is a prerequisite for proper treatment. The current therapy includes medical treatment or a combination of medical treatment and surgery. Prevention by vaccination is not presently available. Mining the genomes of the most common causative agents could identify potential vaccine candidates. Currently, actinomycetoma is treated with antibacterials. The most common drug treatment for uncomplicated cases is trimethoprim-sulfamethoxazole (TMP-SMX) given for several months until cure is achieved [66], [67]. A study in the 1970s by Mahgoub evaluated several antimicrobial combinations in 144 patients with actinomycetoma. In this study, the best clinical outcome was obtained with sulfamethoxazole plus streptomycin. This resulted in the following clinical outcomes: 63.2% cured, 21.5% greatly improved, and 11.1% showing some improvement [68]. In Sudan, the reported cure rate was 43.3%, excluding patients who did not complete the treatment [67]. At present, in Mexico, when there is no therapeutic response or improvement with TMP-SMX, or when the infection is severe and disseminated, or in a special location with a risk of spread to underlying vital tissues (head, neck, trunk, abdomen, or inguinal region), a combination of amikacin (15 mg/kg/day) for 3 weeks plus trimethoprim-sulfamethoxazole (8/40 mg/kg/day) for 5 weeks (a cycle of treatment) is given for up to five cycles. This treatment has achieved a cure rate of >90% [66].

For eumycetoma, antifungal therapy consists mainly of ketoconazole or itraconazole combined with surgical excision [67]. Unfortunately, this therapy is not quite successful. A recent study conducted at the Mycetoma Research Centre in Khartoum, Sudan showed that of the 1,242 eumycetoma patients studied, only 321 (25.9%) were cured, 35 (2.8%) had amputations, and 671 of the patients (54%) dropped out from the outpatient follow-up for various reasons. One of these reasons was the dissatisfaction with the therapy outcome [67]. In addition, in Sudan, most patients pay for their treatments. With a monthly income of $60, the costs of a ketoconazole ($30/month) or itraconazole treatment ($330/month) are a severe financial burden on an average household. Furthermore, it has been known to cause important side effects, such as hepatic toxicity, gynaecomastia, and skin discoloration [69], [70]. Many patients stop therapy and are lost to follow-up in outpatient clinics, only to present later with a massive relapse, often requiring amputation.

For actinomycetoma, 168 out of 302 patients seen in the Mycetoma Research Centre in Khartoum did not complete their treatment. For eumycetoma, the corresponding figures were 671 out of 1,241 patients [67]. Therefore, there is an urgent need to develop better antifungal therapy, better treatment strategies, and a shorter treatment regime. A first step in improving therapy has been made by determining the in vitro susceptibilities of the most common fungal causative agent *M. mycetomatis* against a large panel of marketed antifungal agents [71]–[75]. *M. mycetomatis* appeared to be most susceptible to the azole class of antifungal agents. Only poor inhibitory concentrations were found for other compounds. There is now a need to link in vitro susceptibility data with clinical outcome. There is also a need to generate minimal inhibitory concentration (MIC) breakpoints for in vitro susceptibility linked to therapeutic susceptibility or resistance.

In order to improve current therapy for *M. mycetomatis* infections, the best approaches are to select safer, newer azoles to which the organism is susceptible or use treatments that combine traditionally used azoles with (newly) available antifungal agents with different targets. Antifungal agents that have been used clinically in the treatment of eumycetoma include ketoconazole [76], itraconazole [77], posaconazole [78], voriconazole [79]–[82], and terbinafin [83]. The association of an azole with terbinafin is the most logical combination to be explored, as these two drugs have a different mode of action. Preliminary data on the use of liposomal amphotericin B are promising in terms of efficacy with good drug penetration (Fahal, personal communication). But since this drug is only available intravenously and is highly expensive, its feasibility in the long term for mycetoma treatment remains questionable. As 65% of patients have a secondary bacterial infection [84], the combination of antifungal and antibacterial treatment should be explored.

In addition to the application of new drug therapies for mycetoma, there is a need to shorten treatment times. It is currently difficult to estimate the duration of antibiotic treatment. Based on clinical and radiological criteria, a cure is defined as complete disappearance of the mass, healing of sinuses, skin returned to normal, bone restored to improved radiological appearance, absence of hyper-reflective echoes and cavities on ultrasound examination, and the absence of grains in needle aspirates [67]. The radiological improvement consists of remolding, absorption of the sclerotic bone, and reappearance of the normal trabecular pattern as well as bone density. A simple, reliable biomarker for cure is urgently needed to assess the response to treatment.

## Conclusion

In order to be able to improve the current management of mycetoma, we have identified knowledge gaps for which research is urgently needed. These include the estimation of the burden of mycetoma in order to focus treatment and care on those locations with the highest prevalence. Furthermore, identification of the natural habitat of causal agents and establishing how they are introduced into subcutaneous tissues could lead to novel interventions, which ultimately will reduce its incidence. The development of methods for early diagnosis and therapeutic monitoring will result in better therapeutic outcomes. Improvement of therapeutic strategies in eumycetoma will also reduce the burden of disease. Now that the key research priorities have been highlighted, it is time to implement them. The WHO has taken an important first step by including mycetoma on the list of NTDs. Further recognition by the international community is needed. Resources need to be made available to design control efforts and improve patient management.

**Key Learning Points**

- Obtaining better incidence and prevalence data is necessary to determine appropriate sites for erecting mycetoma health care facilities.
- Gaining insight into the natural habitat and the mode of transmission of the mycetoma causative agents will open the door to preventive measures to reduce the number of mycetoma cases worldwide.
- Developing new, fast, and cheap diagnostic tools will enable early case detection, thereby enhancing the change of a better therapeutic outcome. Furthermore, these tools might also be able to monitor the therapeutic outcome.
- Especially for the treatment of eumyceotma, new therapeutic options are desperately needed.

**Top Five Papers**

1. Abbott P (1956) Mycetoma in the Sudan. Trans R Soc Trop Med Hyg 50: 11–24; discussion, 24–30.
2. Ahmed AO, Mukhtar MM, Kools-Sijmons M, Fahal AH, de Hoog S, et al. (1999) Development of a species-specific PCR-restriction fragment length polymorphism analysis procedure for identification of *Madurella mycetomatis*. J Clin Microbiol 37: 3175–3178.
3. van de Sande WWJ, Luijendijk A, Ahmed AO, Bakker-Woudenberg IA, van Belkum A (2005) Testing of the in vitro susceptibilities of *Madurella mycetomatis* to six antifungal agents by using the sensititre system in comparison with a viability-based 2,3-bis(2-methoxy-4-nitro-5-sulfophenyl)-5- [(phenylamino)carbonyl]-2H-tetrazolium hydroxide (XTT) assay and a modified NCCLS method. Antimicrob Agents Chemother 49: 1364–1368.
4. Welsh O, Vera-Cabrera L, Welsh E, Salinas MC (2012) Actinomycetoma and advances in its treatment. Clin Dermatol 30: 372–381.
5. Mahgoub ES, Gumaa SA (1984) Ketoconazole in the treatment of eumycetoma due to *Madurella mycetomii*. Trans R Soc Trop Med Hyg 78: 376–379.
